# Cadmium Increases Human Fetal Germ Cell Apoptosis

**DOI:** 10.1289/ehp.0900975

**Published:** 2009-10-14

**Authors:** Gaëlle Angenard, Vincent Muczynski, Hervé Coffigny, Catherine Pairault, Clotilde Duquenne, René Frydman, René Habert, Virginie Rouiller-Fabre, Gabriel Livera

**Affiliations:** 1 Laboratory of Differentiation and Radiobiology of the Gonads, CEA–DSV/iRCM/SCSR, Fontenay aux Roses, France; 2 Université Paris Diderot-Paris 7, Fontenay aux Roses, France; 3 INSERM, Unité 967, Fontenay aux Roses, France; 4 Service de Gynécologie-Obstétrique, Université Paris Sud, UMR 782, Hôpital Antoine Béclère, Clamart, France; 5 INSERM, Unité 782, Clamart, France

**Keywords:** cadmium, development, germ cells, human fetus, ovary, testis

## Abstract

**Background:**

Cadmium (Cd) is a common environmental pollutant and a major constituent of tobacco smoke. Adverse effects of this heavy metal on reproductive function have been identified in adults; however, no studies have examined its effects on human reproductive organs during development.

**Objectives:**

Using our previously developed organ culture system, we investigated the effects of cadmium chloride on human gonads at the beginning of fetal life, a critical stage in the development of reproductive function.

**Methods:**

Human fetal gonads were recovered during the first trimester (7–11 weeks postconception) and cultured with or without Cd. We used different concentrations of Cd and compared results with those obtained with mouse fetal gonads at similar stages.

**Results:**

Cd, at concentrations as low as 1 μM, significantly decreased the germ cell density in human fetal ovaries. This correlated with an increase in germ cell apoptosis, but there was no effect on proliferation. Similarly, in the human fetal testis, Cd (1 μM) reduced germ cell number without affecting testosterone secretion. In mouse fetal gonads, Cd increased only female germ cell apoptosis.

**Conclusions:**

This is the first experimental demonstration that Cd, at low concentrations, alters the survival of male and female germ cells in humans. Considering data demonstrating extensive human exposure, we believe that current environmental levels of Cd could be deleterious to early gametogenesis.

The heavy metal cadmium (Cd) is a common environmental pollutant associated with many modern industrial processes. Exposure to this toxicant is usually the result of environmental contamination by waste from human activities, such as the residues found in mining waste, those released by the combustion of fossil fuels and industry, and the runoff from agricultural land (Martelli et al. 2006; Nordberg et al. 1992). Cd occurs in nature at low concentrations; however, its widespread occurrence means that it is present in almost everything that we eat, drink, and breathe (Henson and Chedrese 2004; Thompson and Bannigan 2008). The estimated dietary Cd intake in European countries is 10–30 μg/day (Nasreddine and Parent-Massin 2002). Tobacco smoke is one of the most common sources of Cd contamination in the general population (Nandi et al. 1969; Zenzes et al. 1995), with an estimated assimilation of 0.2–1.0 μg Cd/cigarette (Elinder et al. 1983; Lewis 1972; Satarug and Moore 2004). Cd has a very long biological half-life of 15–30 years (Henson and Chedrese 2004), primarily because of its low rate of excretion from the body, and accumulates over time in the blood, kidney, and liver, where it has numerous undesirable effects on health. In addition, a wide spectrum of deleterious effects on reproductive tissues has also been described (Henson and Chedrese 2004; Piasek and Laskey 1994; Tam and Liu 1985).

Because of its effects on gametogenesis and steroidogenesis, Cd is detrimental to both male and female gonads in adults. Such gonadal alterations have been reported in a variety of animal models, particularly rats and sheep. Male rats have been shown to develop rapid and long-lasting damage in the testes after administration of high doses of Cd (El-Ashmawy and Youssef 1999; Kotsonis and Klaassen 1977). In another study, testicular morphology was greatly altered 3 months after initial Cd exposure, with degenerated seminiferous tubules, abnormal Leydig cells, fibrosis, and reduced testicular size (Niewenhuis 1980). Repeated injections of low doses of Cd also impair spermatogenesis. Furthermore, evidence suggests that Cd alters all testicular germ cell populations. This includes a decrease in the number of spermatogonia and spermatocytes (Aoyagi et al. 2002; Zhou et al. 2004), failure in spermiation (Hew et al. 1993), and compromised viability of spermatozoa (Leoni et al. 2002). Moreover, *in vitro* studies have shown that Cd induces ovine gamete dysfunction. Indeed, 20 μM Cd significantly decreased the viability of spermatozoa, and 2 μM Cd affected their physiologic function (Leoni et al. 2002). Other studies have suggested that subfertility after Cd administration might result from damage to supporting testicular tissue (Bench et al. 1999; Dixon et al. 1976). Elsewhere, Cd was also found to lead to the disruption of the blood–testis barrier (Chung and Cheng 2001; Wong et al. 2004). Finally, Cd treatment of male rats led to a decrease in testicular and plasma testosterone levels (Amara et al. 2008).

In the same way, oocyte development and associated events are disrupted by Cd administration in several species. Rats show dose- and age-dependent toxicity in the ovaries, uterus, and cervix. Cd administration profoundly alters ovarian steroidogenesis (Paksy et al. 1989, 1997a, 1997b; Piasek and Laskey 1994; Zhang et al. 2008) associated with a reduction in progesterone secretion. Similarly, exposure of cultured human (Paksy et al. 1997a) and rat (Zhang and Jia 2007) ovarian granulosa cells to Cd causes a reduction in progesterone production. Cd has also been shown to increase the rate of oocyte degeneration in sheep and impair oocyte maturation in sheep (Leoni et al. 2002) and pigs (Vrsanska et al. 2003).

Only one study has been published on the effect of Cd on fetal gonads (Tam and Liu 1985). In Cd-exposed mice, the authors found reduced genital ridge size in addition to a retarded germ cell migration into the ridges, resulting in depleted germ cell populations, defective maturation of gametes, and subfertility in male offspring.

Despite the growing body of evidence of Cd’s reproductive toxicity and data demonstrating extensive human exposure, no studies have examined the effects of this environmental pollutant on human reproductive development. The fetal stage is critical in the development of reproductive function because the number of germ cells formed during fetal life is related to adult fertility. In males, the two major functions of the testis (i.e., gametogenesis and steroidogenesis) take place during this period. Androgens and insulin-like factor 3, produced by fetal Leydig cells, control the masculinization of the reproductive tract and genitalia (Jost et al. 1973; Kubota et al. 2002). In females, the pool of primordial follicles at birth determines adult fertility, with the depletion of the oogonia stock inducing premature ovarian failure (Mazaud et al. 2002).

In the present study we specifically focused on the effects of Cd on human fetal gonads. We used the fetal gonad organ culture system previously developed by our group coupled with morphologic, functional, and molecular methods (Lambrot et al. 2006a, 2006b, 2007; Livera et al. 2006). Because the effects of *in vivo* Cd exposure have been described previously in mice treated prenatally with Cd (Tam and Liu 1985), we used organ cultures of mouse gonads as our control.

We analyzed the effects of Cd on the development of germ cells in humans during the first trimester of pregnancy (7–11 weeks postconception). This early developmental period is critical for the determination of the reproductive tract in males (Welsh et al. 2008) and for female fertility. We first investigated the effects of Cd on premeiotic female fetal germ cells and measured germ cell apoptosis, then analyzed the effects of Cd on the development of testicular germ cells.

## Materials and Methods

### Collection of human fetal testes

Human fetal gonads were harvested from material available after legally induced abortions in the first trimester of pregnancy (i.e., from the 7th until the 12th week postconception) in the Department of Obstetrics and Gynecology at the Hôpital Antoine Béclère, as previously described (Lambrot et al. 2006a, 2006b). None of the terminations were for reasons of fetal abnormality, and all fetuses appeared morphologically normal. The sex of the fetus was determined from the morphology of the gonads. Ovaries could be distinguished from testes because they are thinner and more closely associated with mesonephros, and a fine blood vessel was detectable on the testes at this fetal stage. In addition, seminiferous cords were visible inside the testes. The fetal age was evaluated by measuring the length of limbs and feet (Evtouchenko et al. 1996). The fetus was dissected under a binocular microscope, and gonads were removed aseptically and immediately explanted *in vitro*. We found gonads within the abortive material in only 20% of cases. The Hôpital Antoine Béclère Ethics Committee approved this study, and all women gave their informed consent.

### Animals

NMRI mice (R. Janvier, Le Genet St-Isle, France) were housed under controlled photoperiod conditions (light from 0800 to 2000 hours) and were supplied with commercial rodent chow and tap water *ad libitum*. Males were caged with females overnight, and the day after an overnight mating was counted as 0.5 day postconception (dpc). Pregnant mice were killed by cervical dislocation on 12.5 dpc, and the fetuses were quickly removed from the uterus. Fetuses were dissected under a binocular microscope, and sex was determined based on the morphology of the gonads. Studies were conducted in accordance with the guidelines on the *Care and Use of Laboratory Animals* issued by the French Ministry of Agriculture (2003); animals were treated humanely and with regard for alleviation of suffering.

### Organ cultures

Human and mouse gonads were cultured on Millicell-CM Biopore membranes (pore size 0.4 μm; Millipore, Billerica, MA, USA) as previously described (Habert et al. 1991; Lambrot et al. 2006a, 2006b; Livera et al. 2006). The culture medium was Dulbecco’s modified Eagle’s medium/Ham F12 (1:1) (Gibco, Grand Island, NY, USA) supplemented with 80 μg/mL gentamicin (Sigma, St. Louis, MO, USA) and 15 mM HEPES. Gonads or pieces of gonads were placed on the membranes floating on 320 μL culture medium in tissue culture dishes and cultured at 37°C in a humidified atmosphere containing 95% air/5% carbon dioxide. Cadmium chloride (Sigma) was dissolved in water (1 M) and stored at −20°C. We measured the responses to Cd (0.1, 1, 10, and 50 μM) either by comparing one gonad cultured in medium containing Cd with the other gonad from the same fetus cultured in control medium (mouse) or by comparing pieces of the same gonad cultured with or without Cd (human).

Culture protocols were optimized according to the species and sex of the gonads. For mice, intact 12.5 dpc gonads were placed on floating filters and cultured for 3–10 days. Each human gonad was cut into small pieces of the same size. For human testes, all pieces from the same gonad were placed on a membrane and cultured for 4 days. For human ovaries, 4–5 pieces from the same gonad were placed on a membrane and cultured for 8 days in the presence of fetal bovine serum (FBS; diluted 1:100). The medium was changed every 24 hr for testes and every 2 days for ovaries. In preliminary experiments we investigated the effect of 1 μM Cd in fetal ovaries cultured with or without FBS (data not shown).The addition of FBS did not change the effect Cd in human (*n* = 2) and in mouse fetal ovaries (*n* = 3).

To determine cell proliferation, we added bromodeoxyuridine (BrdU; 30 μg/mL; 1%; Amersham Biosciences, Little Chalfont, Bucks, England) during the last 3 hr of culture. At the end of the culture, explants were fixed for 2 hr in Bouin’s fluid and embedded in paraffin before being cut into 5-μm sections.

### Germ cell counting

We mounted serial sections on slides, removed the paraffin, and rehydrated the sections. For testes, we performed immunohistochemical assays for anti-Müllerian hormone (AMH), as previously described (Lambrot et al. 2006a, 2006b), using an anti-AMH polyclonal antibody (provided by N. Di Clemente). We then counted germ cells identified as AMH-negative cells within the seminiferous cords. For ovaries, we counted oogonia and oocytes in sections stained with hematoxylin and eosin. The oogonia were identified by their characteristically large size and spherical or near-spherical shape. The interphase nuclei contained fine threads and two or more prominent nucleoli, and the cytoplasmic membrane was clearly visible.

Counting was performed as previously described and validated for rodents (Lambrot et al. 2006a, 2006b; Livera et al. 2000a, 2000b; Olaso et al. 1998) and humans (Guerquin et al. 2009; Lambrot et al. 2006a, 2006b, 2007). For human gonads, we counted germ cells in 1 of 10 sections distributed equidistantly along the pieces of testis for the 7-week-old fetuses and 1 of 20 sections for later stages. For mouse gonads, all germ cells present in 1 of 3 sections were counted. We multiplied the sum of the values obtained for the observed sections of one gonad by 10 or 20 for human testes of fetal ages 7 weeks or > 7 weeks, respectively, and by 3 for mouse gonads to obtain a crude count (CC) of germ cells per gonad. We then used the Abercrombie formula (Abercrombie 1946), which uses the average measured diameter of the germ cell nuclei (*D*) and the thickness of sections (*S*) to correct for any double counting due to single cells appearing in two successive sections, to obtain the true count (TC): *TC* = *CC* × *S*/(*S*+*D*). For human fetal ovaries, germ cell density was measured by dividing the germ cell number by the area of the counted section, as previously described (Guerquin et al. 2009). At least 60 fields were counted from three different sections. All counts were carried out blind and were done using Histolab analysis software (Microvision Instruments, Evry, France).

### Immunohistochemical staining for cleaved caspase-3 and caspase-9

Because caspase-3 is involved in most apoptotic pathways (Omezzine et al. 2003), we used its immunodetection to quantify the rate of apoptosis, as previously described (Delbes et al. 2004; Lambrot et al. 2006a, 2006b). We used similar protocols to detect cleaved caspase-3 and caspase-9, an upstream activator of caspase-3 in the mitochondrial apoptotic pathway. We mounted six sections on a single slide and heated the slide for 30 min in a permeabilization solution (0.05 M Tris, pH 10.6). The primary antibodies—rabbit anticleaved caspase-3 Asp 175 (1/100; Cell Signaling, Beverly, MA, USA) and rabbit anticleaved caspase-9 Asp 353 (1/100; Cell Signaling)—were detected using biotinylated goat antirabbit secondary antibodies in 5% normal goat serum and avidin–biotin–peroxidase complex (Vectastain Elite ABC kit; Vector Laboratories, Burlingame, CA, USA). Peroxidase activity was visualized using 3,3′-diaminobenzidine (DAB) as substrate. For negative controls for all immunohistochemical assays, the primary antibody was omitted.

### Measurement of BrdU incorporation index

BrdU incorporation into proliferating cells was detected by immunocytochemistry, as previously described (Lambrot et al. 2006a, 2006b; Livera et al. 2000a). The BrdU incorporation index was obtained by a blind counting of stained and unstained germ cell nuclei in all sections. At least 500 nuclei were counted for each experiment.

### Testosterone radioimmunoassay

We measured the testosterone secreted into the medium in duplicate by radioimmunoassay, as previously described (Habert et al. 1991). No extraction or chromatography was performed because 17β-hydroxy-5α-androstan-3-one (DHT), the only steroid that significantly cross-reacts with testosterone (64%), is secreted in minute amounts by the fetal testis (George et al. 1987).

### Statistical analysis

All values are expressed as mean ± SEM. For studies on proliferation or apoptosis, we evaluated the significance of the difference between mean values for treated and untreated testes from the same fetus using Wilcoxon’s nonparametric paired test (for small samples). For total germ cell number and density counting, the Student paired *t*-test was used because of the high variability in the number of germ cells between ages. Concerning testosterone secretion analysis, we used one-way analysis of variance to assess the significance of the difference in secretion evolution between control and treated testes during the 3 days of culture.

## Results

### Effect of Cd on female germ cells

**Figure 1:**
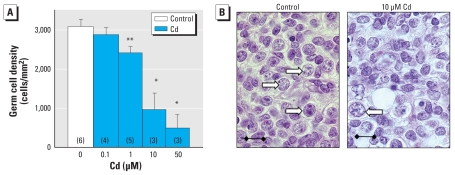
Effect of Cd on germ cell density in the human fetal ovary in culture. (*A*) Germ cell density, determined by counting germ cells in several sections and then normalizing the values with respect to the area. Values shown are mean ± SEM of three to six sections, and numbers in parentheses indicate the number of gonads analyzed. (*B*) Histologic appearance of human fetal ovaries after culture; white arrows indicate germ cells. Bars = 15 μm. See “Materials and Methods” for details of experiments. *p < 0.05, and **p < 0.01 in paired Student’s *t-*test.

After explantation, we cultured human fetal ovaries for 8 days with various concentrations of Cd ranging from 0.1 to 50 μM and then counted the number of germ cells. The six ovaries used in this study ranged in age from 7 to 11 weeks postconception and contained only oogonia. After 8 days, very few (< 0.5%) or no germ cells had initiated meiosis. In control medium, germ cell density was maintained at around 3,000 germ cells/mm^2^ (vs. 2,420 ± 234, mean ± SEM; *n* = 4 before explantation at the same age). Concentrations of Cd ≥ 1 μM significantly and concentration-dependently decreased germ cell density (Figure 1A,B). At 50 μM, Cd frequently induced large areas of necrosis, a phenomenon not observed at lower concentrations.

**Figure 2:**
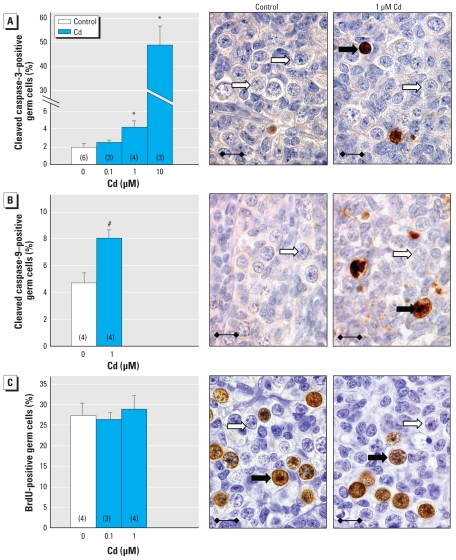
Effect of Cd on the rate of apoptosis and proliferation in human female germ cells cultured with or without Cd. Late and early germ cell apoptosis was measured by immunohistochemical staining of cleaved caspase-3 (*A*) and cleaved caspase-9 (*B*), respectively. Germ cell proliferation was determined by BrdU incorporation (*C*). In photomicrographs of ovaries cultured with 1 μM Cd (*A*, *B*, *C*), black arrows indicate germ cells immunostained for cleaved caspase-3, cleaved caspase-9, or BrdU, and white arrows indicate unstained germ cells. Values shown are mean ± SEM of three to six sections (number shown in parentheses in each bar). Bars = 15 μm. See “Materials and Methods” for details of experiments. *p < 0.05, and ^#^*p* < 0.001 in paired statistical comparisons with the corresponding control values.

Apoptosis was measured by immunodetection of cleaved caspase-3 after culturing (Figure 2A). Hardly any somatic cells were stained for cleaved caspase-3 either in the control or Cd-treated gonads analyzed. In control gonads, very few cells were labeled. Treatment with 1 μM Cd more than doubled the percentage of caspase-3–positive germ cells (1.9 ± 0.5% vs. 4.2 ± 0.8%; mean ± SEM), and 10 μM Cd increased the percentage of stained germ cells by approximately 2,000%; However, no statistically significant change was observed with 0.1 μM Cd. Exposure to 1 μM Cd also doubled the percentage of cleaved caspase-9–positive germ cells (Figure 2B).

We assessed proliferation by BrdU incorporation (Figure 2C). In ovaries cultured in control medium, about one-fourth of germ cells were strongly stained, whereas somatic cells tended to have a very low proliferation rate. Treatment with 0.1 and 1 μM Cd did not change the percentage of oogonia incorporating BrdU, nor did 10 μM Cd analyzed in one gonad (27.5% proliferation rate).

**Figure 3:**
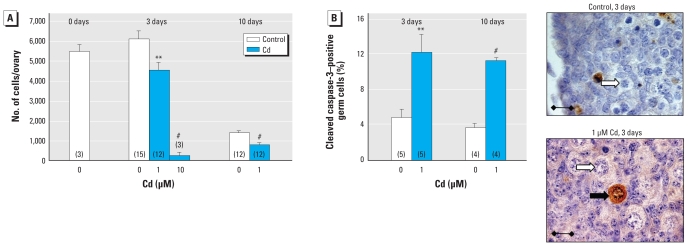
Effect of Cd on germ cell density and apoptosis in mouse fetal ovaries from 12.5 dpc fetuses cultured for 3 days or 10 days in the presence or absence of Cd (0.1, 1, or 10 μM). (*A*) Number of germ cells identified on the basis of morphologic analysis. (*B*) Apoptosis measured by immunohistochemical staining of cleaved caspase-3. In photomicrographs, the black arrow indicates a germ cell immunostained for cleaved caspase-3, and white arrows indicate unstained germ cells. Values shown are mean ± SEM of 3–15 values (number shown in parentheses in each bar). Bars = 15 μm. See “Materials and Methods” for details of experiments. **p < 0.01, and ^#^*p* < 0.001 in paired statistical comparisons with the corresponding control values.

Mouse fetal ovaries were explanted on 12.5 dpc; at this stage, each gonad contained around 5,000 oogonia (Figure 3A). The gonads were then cultured with or without 1 or 10 μM Cd for 3 days, by which time, in control ovaries, the germ cell number was maintained and about 95% of the germ cells had initiated meiosis (mostly leptotena and zygotena stages). Treatment with 10 μM Cd almost fully extinguished the germ cell population, whereas 1 μM Cd induced a 26% decrease in germ cell number. No major change was observed in the distribution of meiotic stages in the presence of Cd (data not shown). After 10 days in culture with or without Cd, all germ cells started forming follicles (data not shown). In control culture, the oocyte number then strongly decreased, with an estimated 1,000 follicles/ovary. After 10 days, 1 μM Cd had decreased the oocyte number to half that of controls.

Few apoptotic germ cells were detected by cleaved caspase-3 staining in mouse fetal ovaries cultured in control conditions (Figure 3B). After 3 and 10 days in culture, 1 μM Cd had strongly and significantly increased the percentage of caspase-3–positive germ cells (2.5- and 3.1-fold, respectively). No BrdU incorporation assays were performed at these stages because meiotic cells had ceased proliferating.

### Effect of Cd on human fetal testes

**Figure 4:**
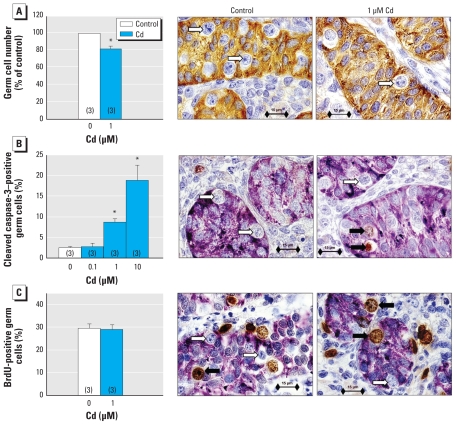
Effect of Cd on proliferation and apoptosis of germ cells in human fetal testes cultured for 4 days with or without Cd (1 μM for total number and proliferation analysis; 0.1, 1, or 10 μM for apoptotic activity analysis) added after the first 24 hr of culture. (*A*) Total number of germ cells per testis (expressed as a percentage of those obtained in the control). (*B*) Apoptosis of the germ cells measured by immunodetection of cleaved caspase-3 (sections of the cultured testes were immunostained for AMH to identify Sertoli cells). (*C*) Proliferating germ cells determined by immunohistochemical detection of BrdU incorporation into the nuclei. Values shown are mean ± SEM of three values. In photomicrographs, black arrows indicate germ cells immunostained for cleaved caspase-3 or BrdU, and white arrows indicate unstained germ cells. Bars = 15 μm. See “Materials and Methods” for details of experiments. *p < 0.05 in paired statistical comparisons with the corresponding control values.

Human fetal testes from 7 to 12 weeks of gestation were cultured for 4 days with or without Cd (0.1, 1, or 10 μM). Immunohistologic analysis of human testes revealed a decrease of about 20% in the total number of germ cells per testis after exposure to 1 μM Cd (Figure 4A). When we measured apoptosis by immunodetection of cleaved caspase-3 after culturing, we observed a 2–8% increase in the apoptotic rate of germ cells (Figure 4B) but no change in their proliferation rate (Figure 4C), which remained at 30%.

**Figure 5:**
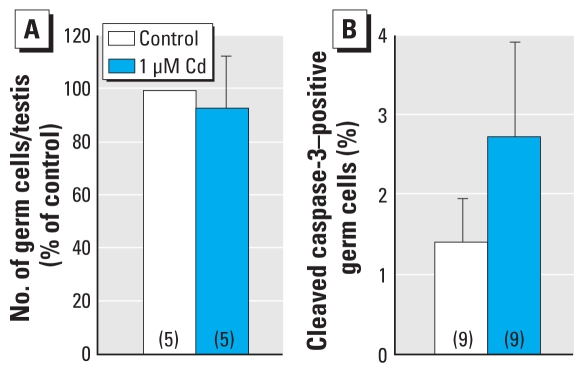
Effect of Cd on the number of germ cells and apoptosis in the mouse fetal testis *in vitro.* (*A*) Number of germ cells identifiedby morphologic analysis. (*B*) Apoptosis measured by immunohistochemical staining of cleaved caspase-3. Values shown are mean ± SEM of five to nine determinations per group (number shown in parentheses in each bar).

To check the possibility of a concentration-dependent effect of Cd toxicity, we measured the rate of apoptosis in the germ cell lineage with increasing exposure to Cd (Figure 4B). With 0.1 μM Cd, the ratio of apoptotic germ cells was similar to that measured in controls; however, with 1 μM and 10 μM Cd we observed increases of 4- and 9-fold, respectively, highlighting a concentration-dependent effect of Cd exposure on the male germ cell line. In the same way, mouse fetal testes (12.5 dpc) were cultured for 3 days in the presence or absence of 1 μM Cd. Immunohistologic analysis of testes showed no significant effect of the addition of Cd on either the total number of germ cells per testis (Figure 5A) or on the rate of apoptosis (Figure 5B) compared with the control. Nevertheless, we observed a trend toward increased apoptosis in Cd-treated testes.

**Figure 6:**
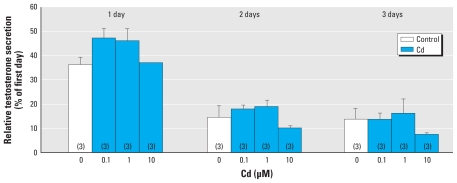
Effect of Cd on testosterone production by human fetal testis *in vitro*. Testes were cultured for 4 days with or without Cd (0.1, 1, or 10 μM) added after the first 24 hr of culture. Testosterone produced on each day was measured by radioimmunoassay; Values (mean ± SEM of three determinations per treatment) are expressed as a percentage of the reference value obtained on day 0.

To test the effect of Cd on steroidogenesis, human fetal testes (7–12 weeks of gestation) were cultured for 4 days with various concentrations of Cd. Testosterone secretion was measured every 24 hr in the culture medium by radioimmunoassay and normalized to the first day. Secretion was not modified by exposure to Cd at any concentration (0.1, 1, or 10 μM) during the 3 days of treatment (Figure 6), nor was it affected in the mouse fetal testis (data not shown).

## Discussion

The heavy metal Cd is a known environmental pollutant and is toxic to reproductive function in different species. In this study, we analyzed the direct effect of Cd on both human male and female reproductive function during fetal life. Our results provide the first demonstration that Cd decreases the germ cell population in developing human gonads.

We used an organotypic culture system developed in our laboratory that has already proven efficient in assessing the toxicity of ionizing radiation and phthalates (Guerquin et al. 2009; Lambrot et al. 2007, 2009). We have thus demonstrated that Cd directly affects human male and female fetal gonads during the first trimester of pregnancy. We have shown that concentrations of Cd as low as 1 μM decrease human germ cell number. This indicates that fetal germ cells are highly sensitive to Cd exposure. In many other human cell types, such as hepatocytes and neuroblastomal cells, Cd triggers deleterious effects only at concentrations > 100 μM (Gulisano et al. 2009; Lasfer et al. 2008). Our observation of a high sensitivity of developing germ cells is highly relevant because 1 μM is only 10–20 times higher than the environmental level of Cd (Satarug and Moore 2004). Moreover, 1 μM Cd decreased fetal germ cell number in only 3 days, whereas the development of human fetal germ cells spans several months. Nevertheless, it should be noted that *in vivo*, Cd is usually bound to serum components such as albumin and metallothionein, whereas in our *in vitro* model, the tissues were exposed to Cd under serum-free conditions (testes) or with very little serum (1% for ovaries).

We demonstrated that Cd decreases both male and female germ cell populations in human fetal gonads. However, when using the same *in vitro* model to assess the effect of Cd in mouse gonads, we observed a similar effect only in mouse ovarian germ cells. Mouse testicular germ cells, on the other hand, seemed unaffected while their rate of apoptosis tended to increase. This difference may be due to a specificity of mouse fetal germ cells that gradually enter mitotic arrest between 12.5 and 14.5 dpc. This quiescence phase is not seen in human germ cells, and we and others have already reported that these resting cells display very little apoptosis (Lambrot et al. 2006a, 2006b, 2007). Altogether, we noted an effect of Cd in human male and female gonads between the 7th and the 12th week postconception and in 12.5 dpc mouse female fetal gonads, with highly mitotic germ cells observed in all stages. We thus hypothesized that Cd mostly affects fetal germ cells during active proliferation. These *in vitro* effects of Cd in mouse and human fetal gonads fit well with the previously described *in vivo* effects of Cd in mice (Tam and Liu 1985). Although this group also reported germ cell depletion in mouse fetal testes, this slight discrepancy may be due to differences in the treatment protocol. We cannot, however, exclude the possibility that the lack of Cd effect observed in mouse testes could be due to an insensitivity of our mouse strain. Indeed, it has been demonstrated that Cd toxicity can vary depending on the strain (Dalton et al. 2005).

We showed here that Cd decreases fetal germ cell number by increasing apoptosis without altering germ cell proliferation. This activation of the apoptotic pathway is highlighted by the increased rate of cleaved caspase-3– and caspase-9–positive germ cells. In the same way, Cd exposure has been reported to induce apoptosis in a primary rat Sertoli cell–gonocyte coculture system (Yu et al. 2008). Moreover, the intrinsic (mitochondrial) pathway is incriminated in Cd-induced apoptosis in many cell types, including C6 rat glioma cells (Watjen et al. 2002). We recently demonstrated that germ cells displayed a sex-specific response to ionizing radiation at stages similar to those used in the present study (Guerquin et al. 2009). Interestingly, although we used a slightly different protocol for culturing testes and ovaries, we observed an almost identical response to Cd in terms of germ cell apoptosis. This would tend to indicate that in germ cells, Cd acts differently from other DNA-damaging agents such as radiation. In addition, Cd has been shown to impair the DNA repair machinery in several cell lines (Giaginis et al. 2006), leading us to consider that such an effect may be the cause of the high sensitivity of proliferating germ cells. Such an effect would also be seriously deleterious for meiotic cells, and indeed we observed that Cd also induced apoptosis in meiotic cells as it also decreased the number of mouse oocytes after meiotic initiation (Figure 3A).

For both males and females, the rate of caspase-3–positive germ cells related to Cd exposure was concentration dependent, with the first significant effect starting with concentrations of 1 μM. Interestingly, 1 μM Cd had no obvious effect on the other gonadal cell types, and the human fetal gonad somatic cells showed no signs of apoptosis. More specifically, in the human testis neither the functioning of Sertoli cells, as revealed by AMH staining, nor that of Leydig cells, as studied via their testosterone production, appeared to be altered by 1-μM Cd treatment. This indicates that Cd probably targets fetal germ cells directly. The direct effect of Cd on isolated human germ cells remains to be determined. Although we report that Cd does not alter testosterone secretion in human or mouse fetal testes, we cannot exclude a possible endocrine-disrupting role of Cd as observed in other vertebrate species. This does, however, appear unlikely, as we and others have been able to show the alteration of rodent testicular testosterone secretion by endocrine disruptors (such as diethylstilbestrol) in organ culture (Lassurguere et al. 2003).

*In vivo*, depletion in the number of germ cells observed in mouse fetal gonads after Cd exposure (Tam and Liu 1985) leads to a defect in germ cell maturation in both sexes and subfertility in males. Although our data rely on an *in vitro* model, the only approach ethically possible with human gonads, one could reasonably hypothesize that similar effects would occur in humans *in vivo*. Thus, effects on female gonads require specific and serious consideration because all female germ cells initiate meiosis during fetal life, giving rise to the definitive stock of oocytes that cannot be renewed later during postnatal life. Therefore, any decrease in female fetal germ cell number may decrease the reproductive life span. In agreement with this notion, we report here that mouse fetal ovaries treated with Cd displayed a reduced stock of follicles after a 10 day culture.

Altogether, our data indicate that human fetal germ cells are highly sensitive to Cd and undergo rapid apoptosis after exposure to low concentrations similar to those occurring in the environment. Our study indicates that both female and male germ cell lineages are roughly equally sensitive to Cd and raises serious concerns about the *in vivo* effects of Cd on human reproduction. Until now, no direct link between Cd exposure during fetal life and subfertility in adulthood has been reported in humans. Relevant future epidemiologic and clinical studies should be made a health priority.
